# A Novel Intraoperative Transient Variant of Immune Thrombocytopenia

**DOI:** 10.7759/cureus.15370

**Published:** 2021-06-01

**Authors:** Abdelghaffar K Salous, Omar Zalatimo

**Affiliations:** 1 General Surgery, Sinai Hospital of Baltimore, Baltimore, USA; 2 Neurological Surgery, Sinai Hospital of Baltimore, Baltimore, USA

**Keywords:** tranexamic acid, thrombocytopenia, idiopathic, immune response, hemostasis

## Abstract

Immune thrombocytopenia, also known as immune thrombocytopenic purpura (ITP) is an autoimmune disorder characterized by a very low number of platelets and associated excessive bleeding. Primary ITP is a diagnosis of exclusion and secondary causes of ITP including lymphoproliferative disorders, medications, and certain infections must be ruled out during workup. This is the first report to highlight intraoperative ITP or an ITP-like novel variant in the perioperative setting leading to increased bleeding. The patient’s extensive workup failed to reveal any secondary causative factors. The clinical presentation of ITP was transient. She received tranexamic acid (TXA), intravenous steroids, and intravenous immunoglobulins (IVIG) and recovered without complication. This case report explores a potentially underreported cause for intraoperative and postoperative hemorrhage in surgical patients.

## Introduction

Intraoperative hemorrhage secondary to immune thrombocytopenic purpura (ITP) is rarely reported. ITP is a heterogeneous disease entity that has been extensively studied and reviewed [1]. ITP can be secondary to lymphoproliferative disorders, certain infections including human immunodeficiency virus (HIV), hepatitis B (HBV), hepatitis C (HCV), or medications. There is no gold standard test for ITP and it remains a diagnosis of exclusion [2]. An extensive review of the available literature to date revealed only one study which showed significant postoperative thrombocytopenia with the nadir still above 100k in non-heparinized orthopedic surgery patients [3]. While corticosteroids, intravenous immunoglobulin (IVIG), and splenectomy have been the cornerstone of treatment [4], other immunosuppressive medications (rituximab), thrombopoietin mimetics, and tranexamic acid (TXA), have emerged as other promising interventions [5]. Here we report a novel and unusual case of idiopathic intraoperative immune thrombocytopenia.

## Case presentation

A 48-year-old Caucasian female presented for operative surgical intervention for neck pain and radiculopathy involving the upper extremity that failed nonoperative treatment. She denied recent or remote symptoms of bruising, overt bleeding, fevers, chills, or infections. Her past medical history included symptomatic cervical stenosis and was negative for hematologic disorders. Her past surgical history included lithotripsy for symptomatic nephrolithiasis, carpal tunnel release, hysterectomy, and uncomplicated pregnancy/delivery. None of these procedures were associated with significant bleeding. However, in her early 20s, the patient underwent a tonsillectomy that was associated with significant hemorrhage and thrombocytopenia, though it did not warrant any additional treatment. Her social history included active smoking and moderate intake of alcohol, but no recreational drug abuse. The patient was adopted with unknown family history. She was not taking any home medications and denied intake of new drugs, herbal supplements, or quinine-containing beverages. Her documented medication allergies included penicillin and Bactrim. Her basic preoperative workup was within normal limits. Her international normalized ratio (INR) was 1.0 and her platelet count was 143 two weeks prior to surgery. 

She presented for her surgical procedure in a stable condition and anesthesia was induced without complications using the routine sequence of medications. Intraoperatively, she received ketamine, lidocaine, famotidine, fentanyl, midazolam, propofol, rocuronium, succinylcholine, clindamycin, lactated ringer’s solution, minimal amounts of pressors epinephrine/phenylephrine, and 8 mg of dexamethasone. She underwent anterior cervical discectomy with fusion (ACDF) followed by posterior percutaneous fusion. Halfway through the surgical procedure, she was noted to have acute onset diffuse bleeding from the muscle, bone, and soft tissues in the wound bed. She did not have hematuria, skin rash, bleeding from mucosal membranes, intravenous access sites, or other defining features of ITP. Several hemostatic agents including Surgiflo^TM^ hemostatic matrix (Ethicon, Somerville, NJ), topical Surgifoam^R^ powder (Johnson & Johnson, Somerville, NJ), 5,000 units of thrombin, and 1 gram of TXA was administered with minimal improvement in the bleeding. Three blood samples obtained intraoperatively approximately 15 minutes apart between each sample, confirmed severe thrombocytopenia with platelet levels of 2000, 2000, and 1000 per microliter, respectively. A peripheral smear was reviewed for both specimens and confirmed severe thrombocytopenia with no clumping or schistocytosis. The patient received two packs of apheresed platelets intra-operatively and a drain was placed in the wound bed. A repeat coagulation studies panel was completely normal.

The patient remained intubated upon transfer to the surgical intensive care unit, and an urgent hematology consultation was requested. Approximately one hour following platelet transfusion, her repeat platelet count was 141k, versus an expected approximate increase of 60k-80k. The rest of her complete blood count showed acute anemia with hemoglobin of ~8.5 (down from 13.2 preoperatively), mild leukocytosis of 13.15, and a differential with neutrophil shift, all of which were consistent with acute postoperative changes. Per the hematologist's recommendation, the patient subsequently received IV methylprednisolone, IVIG, subcutaneous B12 vitamin, and IV folic acid postoperatively despite rebound to normal platelet count. On postoperative day 1, she continued to receive IV steroids and IVIG, and one unit of packed red blood cells. Her hematological workup showed a normal prothrombin time (PT), normal partial thromboplastin time (PTT), INR, normal haptoglobin, slightly decreased fibrinogen activity of 196 (normal>214), mild derangements in the iron panel (in line with acute surgical intervention), negative HIV, HBV, HCV tests, negative antinuclear antibody (ANA) titer, and lack of hepatosplenomegaly or lymphadenopathy on CT imaging of the abdomen/pelvis. She was later extubated, had an unchanged neurologic exam, and was discharged in a stable condition on iron tablets. On follow-up a month later, her preoperative neck pain and radiculopathy resolved.

## Discussion

The presentation, workup, and clinical course of the patient, in this report, lend strong support to the conformity of this clinical presentation to primary ITP. The exceedingly low platelet count was verified with two samples collected approximately 30 minutes apart and a potential laboratory error was excluded by the peripheral smear analysis. This is further supported by the several-fold drop in the hemoglobin requiring red blood cell transfusion. It was also supported by extensive bruising on all dependent areas of her body. It is most interesting that the platelet count rebounded to the pre-operative baseline level of 141k rather than to the appropriately correct level of 60k-80k after the transfusion of only two packs of platelets, even with the administration of one intraoperative dose of dexamethasone and TXA, and prior to any other treatment for ITP (IV Solu-Medrol and IVIG).

This increase out of proportion of the treatment and platelet transfusion indicates a transient variant of ITP. While ITP tends to be more self-limited in pediatric patients, most epidemiologic studies suggest the need for treatment varies proportionally with age [6]. The transient ITP in this patient stands in stark contrast to these epidemiologic trends and suggests a novel ITP variant potentially triggered by the pro-inflammatory milieu of the surgical procedure or the administration of routine medications. Theoretically, while the normalization of the platelet count is multifactorial, its occurrence after intraoperative administration of TXA, prior to the administration of IVIG, in this patient, provides an exciting possibility for the role of TXA in treating drug-induced ITP. This finding falls in line with those by Mayer et al. [5]. However, a recent Cochrane review concluded that the evidence for TXA in treating drug-induced ITP is limited and conclusive results are currently pending completion of ongoing clinical trials [5,7]. With respect to this patient and her prior similar presentation during tonsillectomy, inquiry about possible Fc-gamma receptor polymorphism that predisposes susceptibility to another episode of transient ITP in the future will be explored [8]. As per the Blood Center of Wisconsin, fentanyl or clindamycin could explain secondary drug-induced ITP in this patient [9]. While this provides an explanation for the patient's presentation, the augmented clinical response to TXA, in addition to platelet transfusion, underscores a very important therapeutic role of TXA in such complications. We recommend evaluating patients for possible ITP intraoperatively or in the postoperative period when faced with atypical hemorrhage early, as this may be a transient phenomenon but with lasting avoidable consequences.

## Conclusions

This is the first case report to highlight several critical findings. First, it showed a novel transient intraoperative ITP variant that resulted in significant acute blood loss requiring blood product transfusion and higher level of care. Second, it supported the role of TXA as a potentially effective therapeutic agent in the treatment of ITP. Third, testing for drugs and polymorphisms predisposing to intraoperative ITP could preemptively enable early recognition of this phenomenon and minimizing morbidity secondary to blood loss. Finally, it is the hope of the authors that this report will encourage other surgeons to explore this possibility when faced with a similar presentation to further characterize and manage this preventable/treatable complication.
